# RNA m^6^A Demethylase ALKBH5 Protects Against Pancreatic Ductal Adenocarcinoma *via* Targeting Regulators of Iron Metabolism

**DOI:** 10.3389/fcell.2021.724282

**Published:** 2021-10-18

**Authors:** Rui Huang, Lin Yang, Zhiwen Zhang, Xiaoding Liu, Yi Fei, Wei-Min Tong, Yamei Niu, Zhiyong Liang

**Affiliations:** ^1^Department of Pathology, State Key Laboratory of Complex Severe and Rare Disease, Molecular Pathology Research Center, Peking Union Medical College Hospital, Chinese Academy of Medical Sciences and Peking Union Medical College, Beijing, China; ^2^Department of Pathology, Institute of Basic Medical Sciences, Chinese Academy of Medical Sciences and Peking Union Medical College, Beijing, China

**Keywords:** pancreatic ductal adenocarcinoma (PDAC), ALKBH5, RNA m^6^A methylation, iron metabolism, FBXL5

## Abstract

Although RNA m^6^A regulators have been implicated in the tumorigenesis of several different types of tumors, including pancreatic cancer, their clinical relevance and intrinsic regulatory mechanism remain elusive. This study analyzed eight m^6^A regulators (METTL3, METTL14, WTAP, FTO, ALKBH5, and YTHDF1-3) in pancreatic ductal adenocarcinoma (PDAC) and found that only RNA m^6^A demethylase ALKBH5 serves as an independent favorable prognostic marker for this tumor. To better understand the molecular mechanism underlying the protective effect conferred by ALKBH5 against pancreatic tumorigenesis, we performed a transcriptome-wide analysis of m^6^A methylation, gene expression, and alternative splicing (AS) using the MIA PaCa-2 stable cell line with ALKBH5 overexpression. We demonstrated that ALKBH5 overexpression induced a reduction in RNA m^6^A levels globally. Furthermore, mRNAs encoding ubiquitin ligase FBXL5, and mitochondrial iron importers SLC25A28 and SLC25A37, were identified as substrates of ALKBH5. Mechanistically, the RNA stabilities of *FBXL5* and *SLC25A28*, and the AS of *SLC25A37* were affected, which led to their upregulation in pancreatic cancer cell line. Particularly, we observed that downregulation of FBXL5 in tumor samples correlated with shorter survival time of patients. Owing to FBXL5-mediated degradation, ALKBH5 overexpression incurred a significant reduction in iron-regulatory protein IRP2 and the modulator of epithelial-mesenchymal transition (EMT) SNAI1. Notably, ALKBH5 overexpression led to a significant reduction in intracellular iron levels as well as cell migratory and invasive abilities, which could be rescued by knocking down *FBXL5*. Overall, our results reveal a previously uncharacterized mechanism of ALKBH5 in protecting against PDAC through modulating regulators of iron metabolism and underscore the multifaceted role of m^6^A in pancreatic cancer.

## Introduction

Pancreatic cancer is a highly malignant carcinoma of the digestive system that affects the global population (Siegel et al., 2021). Pancreatic ductal adenocarcinoma (PDAC) is the most common type of all malignant pancreatic carcinomas. No apparent improvements have been observed in patient survival (Mizrahi et al., 2020), despite the acquisition of knowledge on the genetic and epigenetic dysregulation pathways in pancreatic cancer, and advances in the diagnostic and therapeutic approaches. Further exploration of the molecular mechanism underlying tumor initiation and progression is vital to achieve the final goal of improving the clinical outcomes of patients with pancreatic cancer.

*N*^6^-methyladenosine (m^6^A) RNA modification affects all stages of the RNA life cycle and regulates gene expression at the co-transcriptional and post-transcriptional levels (Zhao et al., 2018). m^6^A modification modulates various types of physiological processes, including hematopoiesis (Lv et al., 2018), neural development (Ma et al., 2018; Wang et al., 2018; Weng et al., 2018), spermatogenesis (Zheng et al., 2013; Hsu et al., 2017; Lin et al., 2017), adipogenesis (Zhao et al., 2014), osteogenic differentiation (Yu et al., 2020), and other essential processes. On the other hand, dysfunctional m^6^A regulators and the resultant fluctuation in m^6^A methylation are often observed in various tumors (Huang et al., 2020; Yang et al., 2020). Accumulating evidence has shown that several m^6^A regulators exert either promotive or inhibitory effects on the hallmarks of cancer, such as cell proliferation, immune evasion, tumor invasion and metastasis (Barbieri and Kouzarides, 2020). Iron is an essential element for various cellular functions while dysregulation of iron metabolism plays a role in tumor progression and metastasis (Torti and Torti, 2020b). However, the existing knowledge on the crosstalk between m^6^A methylation and iron metabolism is extremely limited. Recent study has identified that YTHDF1 accelerates the tumorigenesis of hypopharyngeal squamous cell carcinoma (HPSCC) via the enhancement of iron metabolism (Ye et al., 2020), while the involvement of other m^6^A regulators in the control of iron metabolism remains unclear.

Previous studies have reported that METTL3 (Xia et al., 2019), METTL14 (Wang M. et al., 2020; Chen S. et al., 2021), WTAP (Li et al., 2019), FTO (Tang et al., 2019), ALKBH5 (Guo et al., 2020; Tang et al., 2020), YTHDF2 (Chen et al., 2017), and YTHDC1 (Hou et al., 2021) play pivotal roles in regulating the proliferation, metastasis, and chemosensitivity of pancreatic cancer cells. However, the underlying mechanism and clinical relevance of these RNA m^6^A regulators remain to be fully elucidated. Although PDAC is the utmost stroma-rich cancer, previous studies were limited to the role of m^6^A in tumor cells, while neglecting the difference between the tumor and stroma. Herein, we evaluated the expression of these RNA m^6^A regulators in tumor cells and stromal cells and their potential prognostic values for PDAC patients. Furthermore, we focused on ALKBH5 for intensive investigation of its molecular mechanism in protecting against pancreatic cancer.

## Materials and Methods

### Pancreatic Ductal Adenocarcinoma Tissue Samples and Tissue Microarrays

Tissue microarrays [formalin-fixed, paraffin-embedded (FFPE)] of PDAC tumor and normal tissue adjacent to tumors (para-tumor) collected between September 2008 and July 2013, together with corresponding hematoxylin and eosin (H&E)-stained slides, were provided by the Department of Pathology, Peking Union Medical College Hospital (PUMCH, Beijing, China). The specimens were histologically diagnosed by two experienced pathologists and staged according to the 8th edition of the American Joint Committee on Cancer TNM Staging System. Clinical and pathological data, including age, sex, tumor location, lymph node invasion, neural invasion, bile invasion, and tumor TNM stage were extracted from medical records with follow-up period ranging from 2 to 54 months. A total of 63 PDAC tumor and 27 para-tumor samples were included in this study, excluding the samples that fell off from the tissue sections. This study was approved by the PUMCH Ethical Committee (JS-1490), and informed consent was obtained from all patients in accordance with the Declaration of Helsinki.

### Immunohistochemical Staining and Evaluation

The PDAC tissue sections (4 μm) were subjected to IHC staining. The sections were deparaffinized with xylene and rehydrated with serial dilutions of ethanol (100, 95, and 75%). Antigen retrieval was performed by heating the sections in a citrate buffer solution (0.01 M, pH = 6.0) at 95°C for 10 min or under high pressure for 2 min 10 s. Subsequently, endogenous peroxidase activity in the tissues was blocked in 3% H_2_O_2_ at ∼25°C for 10 min. The slides were sequentially incubated with primary antibodies and horseradish peroxidase (HRP)-labeled secondary antibodies (Supplementary Table 1). Finally, the slides were stained with diaminobenzidine (DAB) and counterstained with hematoxylin.

The expression of each individual gene in the normal pancreatic ductal epithelial cells, tumor cells, or stromal cells was scored separately using the H-score. The intensity and percentage of the positive cells were scored independently by two pathologists. The H-score represents the sum of the intensity of each stain (grades 0–3, where 0, 1, 2, and 3 represent negative, weak, moderate, and strong staining) multiplied by the percentages of the cells positive for each marker (0–100%). The final H-score can range from 0 to 300.

### Construction of MIA PaCa-2 Stable Cell Line Constitutively Expressing ALKBH5

The human pancreatic cancer cell line MIA PaCa-2 was obtained from the Cell Resource Centre of Peking Union Medical College (Beijing, China). The cells were cultured in high-glucose Dulbecco’s modified Eagle’s medium (DMEM) (Corning, 10-017-CV) supplemented with 10% fetal bovine serum (FBS) (Corning, 35-010-CV) in an incubator at 37°C in the presence of 5% CO_2_. The lentiviruses expressing empty vector (EV) and N-terminal Flag-tagged ALKBH5 (NM_017758) were purchased from OBiO Technology (Shanghai, China). MIA PaCa-2 was transduced with lentiviruses and selected via the limited dilution assay.

### Methylated RNA Immunoprecipitation (m^6^A-IP) and Sequencing (m^6^A-Seq)

The total RNA was extracted from EV and ALKBH5-overexpressing (OE) MIA PaCa-2 cells by using TRIzol^TM^ Reagent (Invitrogen, 15596026), according to the manufacturer’s instructions. Poly(A) RNA was isolated from the total RNA using the poly(A) Spin^TM^ mRNA Isolation Kit (NEB, S1560). Poly(A) RNA was fragmented into ∼ 200 nt using RNA Fragmentation Reagents (Ambion, AM8740). A total of 1 μg of fragmented poly(A) RNA was employed for m^6^A-IP, which was achieved using the Magna MeRIP^TM^ m^6^A Kit (Millipore, 17-10499). Immunoprecipitated RNA was recovered with the RNeasy MinElute^®^ Cleanup Kit (Qiagen, 74204). The cDNA libraries were prepared with the NEBNext^®^ Ultra^TM^ RNA Library Prep Kit for Illumina^®^ (NEB, E7530L). Next-generation sequencing was conducted on the Illumina X Ten platform.

The total RNA purified from the EV and OE MIA PaCa-2 cells was fragmented into ∼100 nt. A total of 10 μg of fragmented total RNA was diluted in 1 × IPP buffer (Tris-HCl, pH = 7.4, 50 mM; NaCl, 750 mM; NP-40, 0.5% vol/vol) and incubated with anti-m^6^A antibody. The m^6^A-enriched RNAs were eluted in 1 × IPP buffer containing m^6^A (BERRY & ASSOCIATES, PR3732) and purified with RNeasy MinElute^®^ Cleanup Kit (Qiagen, 74204). The cDNA libraries were constructed using the SMARTer^®^ Stranded Total RNA-Seq Kit v2 - Pico Input mammalian (Takara, 634414). Next-generation sequencing was conducted on the Illumina NovaSeq 6000 platform.

The m^6^A level of a specific gene was detected using m^6^A-IP, which was performed with 10 μg of fragmented total RNA (∼400 nt) along with 0.1 fmol of negative and positive spike-in control RNA, unmodified *Cypridina Luciferase* control RNA (*Cluc*) and m^6^A-modified *Gaussia luciferase* RNA (*Gluc*), provided within the EpiMark^®^ *N*^6^-methyladenosine Enrichment Kit (NEB, E1610S).

### m^6^A-Seq Data Analysis

The m^6^A-seq data were analyzed in accordance with the procedures described in a previous study (Chang et al., 2017). The clean reads of each sample were mapped against the human genome (version hg38). Only uniquely mapped reads were included in the subsequent analyses. The gene expression levels were evaluated using the reads per kilobase of transcript per million mapped reads (RPKM) values. The genes that were expressed differentially (|log_2_FC| > 0.58, *P* < 0.05) between the EV and OE samples were identified using the *edgeR* (McCarthy et al., 2012) or *DESeq2* R package (Love et al., 2014). The *exomePeak* R package (Meng et al., 2013) was used to identify the RNA m^6^A-modified regions (m^6^A peaks) in each sample, and HOMER (Heinz et al., 2010) was used to determine the conserved motifs within these regions. We divided the 3′UTR, coding sequence region (CDS), and 5′UTR regions of the longest transcript of each gene into 100 equally sized bins, respectively, to characterize the distribution patterns of m^6^A peaks. The percentage of m^6^A peaks in each bin was calculated to represent the occupancy of m^6^A along with the transcripts. Differentially methylated regions (DMRs) between the EV and OE samples were further identified using *exomePeak* software by taking the cutoff of |log_2_FC| > 0.58 and false discovery rate (FDR) < 0.05. The Gene Ontology (GO) analysis of the differentially expressed or modified genes was conducted based on DAVID online annotation database (Huang da et al., 2009a,b). The visualization of the enriched GO terms was implemented using the *ggplot2* R package.

### Detection of Alternative Splicing Events

The input RNA-seq data obtained from the EV and OE samples were utilized to detect alternative splicing (AS) events using the replicate multivariate analysis of transcript splicing (*rMATS*) tool (Shen et al., 2014). This tool enables the detection of 5 types of AS events: alternative 5′ splicing site (A5SS), alternative 3′ splicing site (A3SS), mutually exclusive exons (MXE), retained intron (RI), and skipped exon (SE). It can also identify the AS event that exhibits significant alterations by comparing the inclusion levels between the samples in different conditions. The inclusion levels of each event were quantified by the percent spliced in (PSI) which was calculated according to the inclusion junction counts (IJC) and skipping junction counts (SJC) in each splicing event. The AS events with a FDR < 0.05 and |ΔPSI| > 0.2 and (IJC + SJC) > 12 in the comparison results were considered as significantly dysregulated AS events.

### RNA Immunoprecipitation

The MIA PaCa-2 cells were collected and lysed in non-denaturing lysis buffer [Tris-HCl, pH = 7.4, 50 mM; NaCl, 250 mM; Triton X-100, 0.5%; dithiothreitol (DTT), 1 mM; ethylenediaminetetraacetic acid (EDTA), 2 mM; NaF, 1 mM; protease inhibitor cocktail, 1×; RNase inhibitor (RNasin), 0.04 U/mL], followed by bicinchoninic acid (BCA) protein quantification (Thermo Scientific, 23227). The whole-cell lysates were incubated with the anti-ALKBH5 antibody at 4°C on a rotator for 5 h, followed by the addition of protein A/G magnetic beads (Thermo Scientific, 26162) to the mixture and overnight incubation at 4°C on a rotator. The magnetic beads were sequentially washed in low salt Tris-buffered saline (TBS) (Tris-HCl, pH = 7.4, 50 mM; NaCl, 250 mM; DTT, 1 mM; NaF, 1 mM; protease inhibitor cocktail, 1×; RNasin, 0.04 U/ml) and high salt TBS (Tris-HCl, pH = 7.4, 50 mM; NaCl, 300 mM; DTT, 1 mM; NaF, 1 mM; protease inhibitor cocktail, 1×; RNasin, 0.04 U/mL), followed by treatment with Proteinase K Buffer [Tris-HCl, pH 7.4, 100 mM; NaCl, 150 mM; EDTA, 12.5 mM; sodium dodecyl sulfate (SDS), 2% w/v; proteinase K, 1.2 mg/mL] at 55°C for 30 min. A total of 10 mL of supernatant was subjected to western blot analysis to determine the efficiency of immunoprecipitation. The remaining supernatant was used for RNA purification with the RNeasy MinElute^®^ Cleanup Kit (Qiagen, 74204).

### RNA Stability Assay

The EV and OE MIA PaCa-2 cells were used for the RNA stability assay. The cells were seeded onto 48-well plates in triplicate. Actinomycin D (Sigma, A4262) was added to each well after 24 h to achieve a final concentration of 5 μg/mL and incubated for 0, 3, 6, and 9 h. The cells were collected, and total RNA was purified using TRIzol^TM^ Reagent (Invitrogen, 15596026).

### Reverse Transcription, Quantitative Real-Time Polymerase Chain Reaction and Polymerase Chain Reaction

The immunoprecipitated RNA, input RNA, and total RNA were reverse-transcribed using the GoScipt^TM^ Reverse Transcription System (Promega, #A5000). The m^6^A-induced changes in specific genes and ALKBH5-associated RNAs were determined via the qPCR using PowerUp^TM^ SYBR^TM^ Green PCR Master Mix (ABI, A25742) on a qPCR instrument machine (Roche, LightCycler^®^ 480 II). The alternatively spliced products were determined with PCR via 2 × GoldStar MasterMix (CWBIO, CW0929L) and agarose gel (2%) electrophoresis. The primers used in the study are listed in Supplementary Table 2.

### Western Blot Analysis

The proteins were purified using the radioimmunoprecipitation assay buffer (RIPA) (APPLYGEN, C1053+) and quantified with the BCA assay. Proteins were separated by 8, 10, or 12% SDS-PAGE and transferred to polyvinylidene fluoride (PVDF) membranes (Millipore, ISEQ00010). The membranes were blocked with 10% non-fat milk for 2 h at ∼25°C, followed by overnight incubation with primary antibodies at 4°C. The membranes were washed with Tris-buffered saline with Tween 20 (TBST) buffer (APPLYGEN, B1009) for 10 min and subsequently incubated with the HRP-labeled secondary antibodies using Chemidoc^TM^ Touch Image System (BIO-RAD, 1708370). The antibodies used in this study are listed in Supplementary Table 1.

### siRNA Transfection

RNA oligos were synthesized by RiboBio Co., Ltd. (Guangzhou, China). The siRNA sequences used are as following: Scramble (SC): 5′-*GGCUCUAGAAAAGCCUAUGC*-3′, si*FBXL5*-1 (KD-1): 5′-*UGCGUAUUGUGGUCACUCA*-3′, si*FBXL5*-2 (KD-2): 5′- *GU UUGCACGAUUUAACUAA*-3′. The siRNAs were transfected into OE MIA PaCa-2 cells with RNAiMax (Invitrogen, 13778150). Forty-eight hours post transfection, cells were collected and proceeded to Western blot analysis, cell migration and invasion assay, and intracellular iron assay.

### Cell Migration and Invasion Assays

Cell migration and invasion assays were performed using transwell chambers (8-μm pore size) (Corning Inc., 3422) with or without Matrigel matrix (Corning Inc., 356234). Cells were resuspended with DMEM and seeded into the upper chambers, and the lower chambers were filled with DMEM supplemented with 10% FBS. After ∼16 h, the non-migrating or non-invading cells were wiped off from the membranes, followed by fixation in 37% formaldehyde and staining with 3% crystal violet solution. Images were captured and then the migrated cells were counted.

### Intracellular Iron Assay

Cellular iron levels were assayed by using commercial Iron Assay Kit (Colorimetric) (Abcam, ab83366), according to the manufacturer’s instructions. Briefly, cells were collected and homogenized in iron assay buffer, the cell lysates were centrifuged at 16,000 × *g* for 10 min to collect the supernatant. Iron probes were added into each sample and incubated at 37°C for 1 h. The absorbance at 593 nm were measured with a colorimetric microplate reader.

### Statistical Analysis

Each experiment was performed in triplicate. Data were presented as the mean ± standard deviation (SD). Statistical analyses were conducted using Graphpad Prism 7 (Graphpad Software Inc., San Diego, CA, United States) and SPSS 22.0 (SPSS Inc., Chicago, IL, United States). Differences between the groups were analyzed using Student’s *t*-test. The survival status was evaluated using Kaplan–Meier curves and the log-rank test. Cox-regression analyses were used to ascertain the independent prognostic factors for PDAC. Two-tailed *P*-values < 0.05 were considered statistically significant.

## Results

### Reduced ALKBH5 Expression Correlates With Poor Prognosis of Patients With Pancreatic Ductal Adenocarcinoma

**FIGURE 1:**
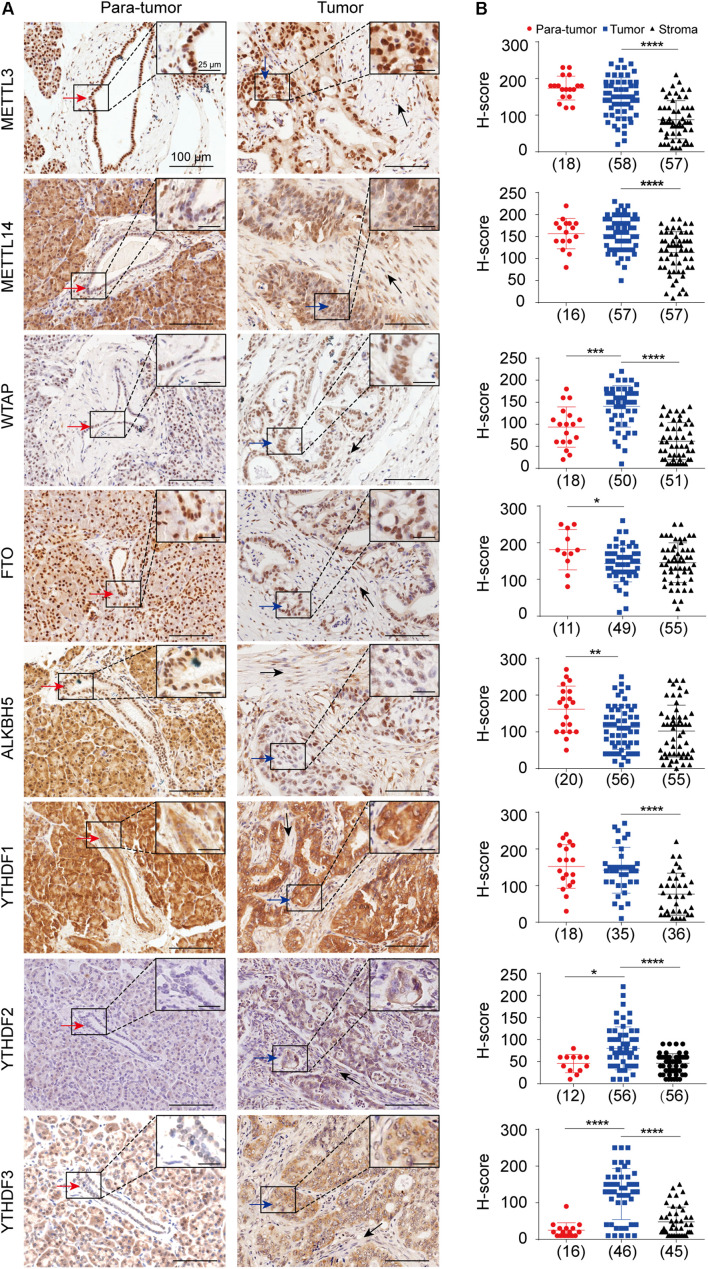
*In situ* protein expression analysis of eight m^6^A key regulators in pancreatic ductal adenocarcinoma (PDAC). **(A)** Representative immuno-histochemical staining showing the expressions of METTL3, METTL14, WTAP, FTO, ALKBH5, and YTHDF1-3 in normal pancreatic ductal epithelial cells (red arrows) in para-tumor samples, tumor cells (blue arrows), and stromal cells (black arrows) in tumor samples. Enlarged images in the box are shown in the upper right. **(B)** Scatter plots showing the H-scores of each gene in para-tumor, tumor and stroma cells are shown in the right panel. Case numbers are shown in brackets. Scale bars, 100 or 25 μm. *P < 0.05, **P < 0.01, ***P < 0.001, ****P < 0.0001.

Pancreatic ductal adenocarcinoma is a kind of epithelial tumor arising from pancreatic ductal cells, which is characterized by the extensive proliferation of stromal cells (Lee et al., 2019; Neesse et al., 2019). Even though the roles of several RNA m^6^A regulators have been identified in pancreatic cancer, their expressions in the tumor cells and stroma cells remain to be thoroughly explored. Herein, we performed IHC analysis by using the TMAs including 63 samples to detect the *in situ* expression patterns of eight m^6^A regulators in the normal pancreatic ductal epithelial cells, PDAC tumor cells, and stroma cells, respectively (Supplementary Figure 1). The methyltransferases and demethylases analyzed in this study were mainly located in the nucleus in both normal epithelial cells and tumor cells, while the m^6^A-binding protein was located in the cytosol (Figure 1A). The tumor cells exhibited a significant increase in the expression of WTAP, YTHDF2, and YTHDF3, but a reduction in the expression of FTO and ALKBH5 compared to the normal epithelial cells in the para-tumor samples. Meanwhile, we found that the expressions of METTL3, METTL14, WTAP, and YTHDF1-3 proteins were significantly lower in the stroma cells than that in the tumor cells (Figures 1A,B).

**FIGURE 2:**
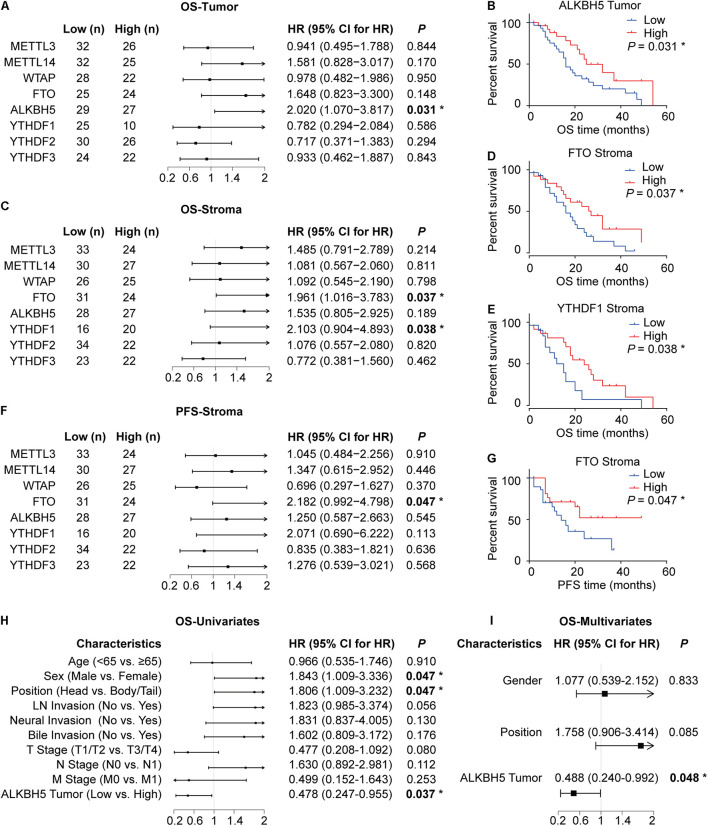
Downregulation of ALKBH5 in PDAC predicts poor prognosis. **(A)** Log-rank analysis of correlation between overall survival (OS) and the expression of each m^6^A regulator in tumor. **(B,C)** Log-rank analysis of correlation between OS or progression-free survival (PFS) and the expression of m^6^A regulators in stroma. **(D–G)** Kaplan–Meier analysis of correlation between OS or PFS and ALKBH5, FTO, and YTHDF1 protein expression. **(H,I)** Cox regression analysis for OS in PDAC patients. *P < 0.05.

We subsequently performed log-rank test to determine the association between the expression levels of each m^6^A regulator and the overall survival (OS) or progression-free survival (PFS) duration to explore the potential clinical relevance of each m^6^A regulator in PDAC. ALKBH5 expression in tumor cells exhibited a positive correlation with OS time in patients with PDAC (Figures 2A,B). In addition, the decreased expressions of FTO and YTHDF1 in the stroma were predictive of a poor prognosis (Figures 2C–G). In contrast, we failed to observe any significant correlation between the expressions of other m^6^A regulators (METTL3, METTL14, WTAP, YTHDF2, and YTHDF3) with patient survival (Figures 2A,C,F and Supplementary Figure 2A). Further univariate and multivariate cox regression analyses showed that out of the eight RNA m^6^A regulators, only ALKBH5 was an independent predictive marker for the prognosis of patients with PDAC (Figures 2H,I and Supplementary Figures 2B–E).

The above-mentioned results collectively illuminate that ALKBH5 is downregulated in PDAC and that, it might be the only m^6^A regulator (among the eight RNA m^6^A regulators identified in this study) capable of predicting the prognosis independently in patients with PDAC.

### m^6^A-Seq Reveals Decreased Methylation Level Upon ALKBH5-Overexpression

Next, we generated an MIA PaCa-2 stable cell line constitutively expressing Flag-ALKBH5 for subsequent analyses to unveil the functions and potential targets of ALKBH5 in PDAC. First, we examined the effect of overexpressed ALKBH5 on RNA m^6^A methylation at transcriptome-wide level based on m^6^A-seq analysis of poly(A) RNA isolated from the EV and OE MIA PaCa-2 cells.

**FIGURE 3:**
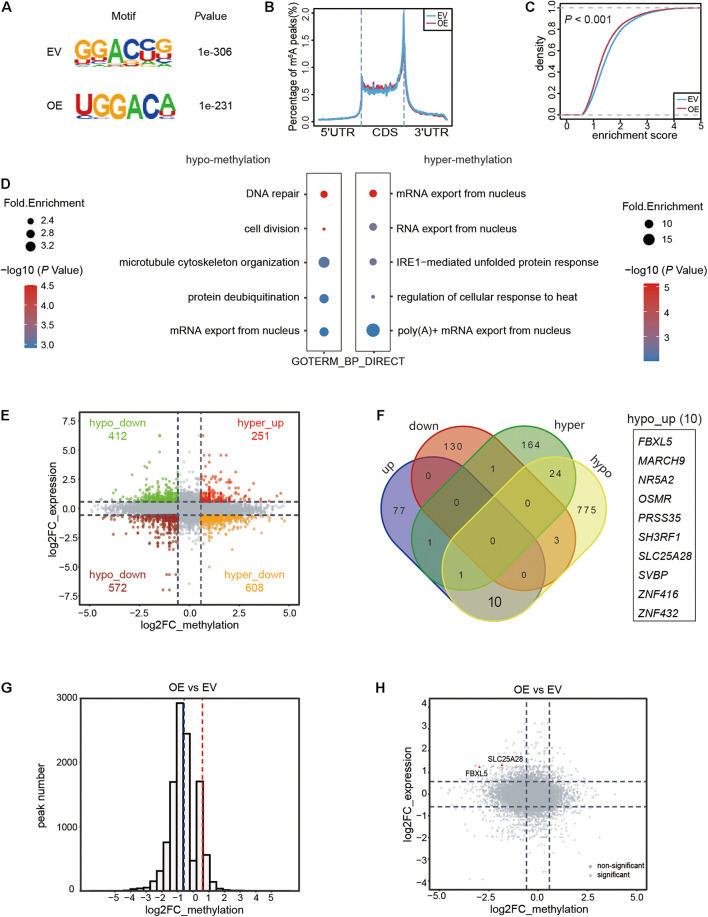
Transcriptome-wide RNA m^6^A methylation and expression analysis of MIA PaCa-2 stable cell line with ALKBH5 overexpression. **(A)** Top consensus motif of m^6^A peaks identified in empty vector (EV) or ALKBH5-overexpressing (OE) MIA PaCa-2 cells. **(B)** Normalized distribution of m^6^A peaks across 5′UTR, CDS, and 3′UTR of mRNAs. **(C)** Cumulative distribution function of log2 enrichment score of m^6^A modified sites. **(D)** GO term analysis of transcripts with hypo- **(left panel)** and hyper-methylated **(right panel)** m^6^A sites in OE sample versus EV sample. **(E)** Scatter plot representing the log_2_FC of methylation (*x*-axis) and expression (*y*-axis) of each m^6^A peak in OE versus EV sample. Points with |log_2_FC| > 0.58 in both differential expression and methylation analysis results are highlighted. **(F)** Venn diagram showing the RNAs with significant change in either methylation (|log_2_FC| > 0.58 and FDR < 0.05) or expression (|log_2_FC| > 0.58 and *P* < 0.05) in OE sample. **(G)** Histogram showing the fold changes in m^6^A enrichment between OE and EV samples from total RNA based m^6^A data. Dotted lines denote the threshold (|log_2_FC| > 0.58) for filtering differential expression gene. **(H)** Scatter plot showing the distribution of fold changes in methylation (*x*-axis) and expression (*y*-axis) of each RNA in OE versus EV sample. Two RNAs with significantly decreased m^6^A methylation and increased expression level in OE sample are marked in red. Points in rectangle shape represent significant changes in methylation (|log_2_FC| > 0.58 and FDR < 0.05) and expression (|log_2_FC| > 0.58, *P* < 0.05), while the rest ones are shaped in circle.

We identified 11,100 and 10,974 m^6^A methylation peaks located in the mRNAs in the EV and OE samples, respectively (Supplementary Table 3). The m^6^A sites were mainly distributed in the GGAC context in both samples (Figure 3A) and located in the CDS, with a significant enrichment in the stop codon region (Figure 3B), which was consistent with previous studies. We found that the global methylation level decreased significantly (*P* < 0.001) in the OE sample (Figure 3C). Using (|log_2_FC| > 0.58, FDR < 0.05) as the criteria, we identified 194 hyper-methylated and 882 hypo-methylated m^6^A peaks in the OE sample, which were distributed in 191 and 813 mRNAs, respectively (Supplementary Table 4). The substantially greater number of hypo-methylated peaks compared to the hyper-methylated peaks was consistent with the function of ALKBH5 as a demethylase enzyme. Functional enrichment analysis of the genes encoded by differentially methylated mRNAs revealed that the genes regulated by ALKBH5 via RNA methylation participated in various functional pathways. The hypo-methylated RNAs in the OE sample were involved in pathways such as DNA repair, cell division and microtubule cytoskeleton organization (Figure 3D). In contrast, the hyper-methylated RNAs in the OE sample were mainly enriched in functions including RNA export, IRE1-mediated unfolded protein response and others. These results imply that ALKBH5 exerts its functions on multiple signaling pathways depending on m^6^A methylation in the pancreatic cancer cell line.

We also observed that the expression levels of some genes were altered upon overexpression of ALKBH5, including 89 upregulated and 134 downregulated protein coding genes (|log_2_FC| > 0.58, *P* < 0.05) in the OE MIA PaCa-2 cells (Supplementary Table 5). Given the pivotal effects of m^6^A methylation in modulating RNA processing, we integrated the RNA methylation and expression data to explore their implications in pancreatic cancer cells. We found that a substantial number of genes harbored both abnormal RNA expression and methylation levels (|log_2_FC| > 0.58) in the OE sample (Figure 3E). Only a few were filtered out after taking the significance of difference (*P* [differential expression] < 0.05 and FDR [differential methylation] < 0.05) into consideration (Figure 3F). Given the nature of ALKBH5 as a demethylase, we prioritized these hypo-methylated RNAs for subsequent analyses. Only three of the hypo-methylated mRNAs exhibited significantly lower (log_2_FC < −0.58 and *P* < 0.05) expression levels (hypo_down), while ten RNAs exhibited increased (log_2_FC > 0.58, *P* < 0.05) expression levels (hypo_up) in the OE MIA PaCa-2 cells (Figure 3F).

The above-mentioned findings were further validated using another two sets of total RNA-based m^6^A-seq using the EV and OE MIA PaCa-2 cells (Supplementary Figures 3A,B), followed by differential methylation and expression analyses (Supplementary Tables 6, 7). Most of m^6^A methylation regions were hypo-methylated upon ALKBH5-overexpression (Figure 3G), consistent with the above-mentioned results. Notably, out of the ten hypo-up RNAs identified in the original sequencing data (Figure 3F), only two RNAs (*FBXL5* and *SLC25A28*) exhibited significantly lower methylation levels and higher expression levels in the OE MIA PaCa-2 (Figure 3H). The above results implied that ALKBH5 overexpression in pancreatic cancer cells induced the overall demethylation of mRNAs. Importantly, we identified two RNAs, *FBXL5* and *SLC25A28*, as potential substrates of ALKBH5, as evidenced by the simultaneous changes in both methylation and gene expression upon ALKBH5-overexpression.

### ALKBH5 Regulates RNA Stability of *FBXL5*

**FIGURE 4:**
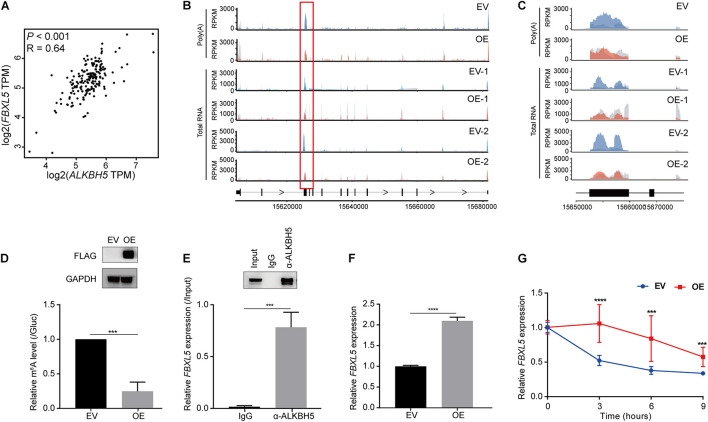
ALKBH5 regulates the stability of *FBXL5* RNA. **(A)** Expression correlation between *ALKBH5* and *FBXL5* across 178 pancreatic adenocarcinoma (PAAD) samples in TCGA database. R, Pearson correlation coefficient. **(B)** Sequencing reads density for input and m^6^A-IP samples along *FBXL5* transcript in poly(A) RNA- and total RNA-based m^6^A-seq data. Hypo-methylated peaks are indicated in red rectangles. Gray for input libraries, while blue (EV) or red (OE) for IP libraries. *X*-axis, genomic coordinates; *Y*-axis, normalized number of reads. **(C)** Zoom-in of the hypomethylated region on *FBXL5* transcript. **(D)** m^6^A-IP-qPCR results showing the methylation changes of *FBXL5* between EV and OE samples. Results of Western blot analysis showing the expression of Flag-ALKBH5 are shown in the upper panel. GAPDH is used as an internal control. **(E)** RIP-qPCR results showing the interaction between ALKBH5 protein and *FBXL5* RNA in MIA PaCa-2 cells. Results of Western blot analysis showing the efficiency of immunoprecipitation are shown in the upper panel. **(F)** RT-qPCR results showing the changes of *FBXL5* expression between EV and OE samples. **(G)** Results of RNA stability assay showing the effect of ALKBH5 overexpression on the decay rate of *FBXL5* RNA. EV, empty vector; OE, ALKBH5 overexpression. ***P < 0.001, ****P < 0.0001.

We subsequently investigated whether the two genes were regulated as direct downstream targets of ALKBH5 in PDAC. First, we performed gene expression correlation analysis using 178 pancreatic adenocarcinoma (PAAD) samples according to the Cancer Genome Atlas (TCGA) database to explore their correlation with *ALKBH5* (Tang et al., 2017). We found that ALKBH5 exhibited a significantly positive correlation with *FBXL5* (*R* = 0.63, *P* < 0.001) (Figure 4A). Therefore, we further investigated the mechanism by which ALKBH5 regulated *FBXL5* RNA dependent on m^6^A demethylation. According to the above-mentioned results of m^6^A-seq, ALKBH5 overexpression led to a significant reduction in the m^6^A levels at the third exon of *FBXL5* in both poly(A) RNA- and total RNA-based data (Figures 4B,C), which was consequently validated by m^6^A-IP-qPCR (Figure 4D). Meanwhile, we also detected a physical interaction between the ALKBH5 protein and *FBXL5* RNA (Figure 4E). On the basis of positive regulation of ALKBH5 on *FBXL5* expression (Figure 4F), we performed an RNA stability assay and found that ALKBH5 overexpression substantially delayed its RNA degradation (Figure 4G). In parallel, we also observed significant decrease in the m^6^A level around the last exon of *SLC25A28* (Supplementary Figures 4A–C). Furthermore, ALKBH5 protein interacted with *SLC25A28* RNA (Supplementary Figure 4D) and affected its expression and RNA stability as well (Supplementary Figures 4E,F). However, despite of the above evidence, *SLC25A28* RNA exhibited a relatively weaker correlation with ALKBH5 in their RNA expression levels in PDAC patients (*R* = 0.21, *P* = 0.006) (Supplementary Figure 4G). Taken together, we show here that *FBXL5* and *SLC25A28* RNAs are potential substrate RNAs regulated by ALKBH5 in their RNA stabilities.

### ALKBH5 Regulates Alternative Splicing of *SLC25A37*

**FIGURE 5:**
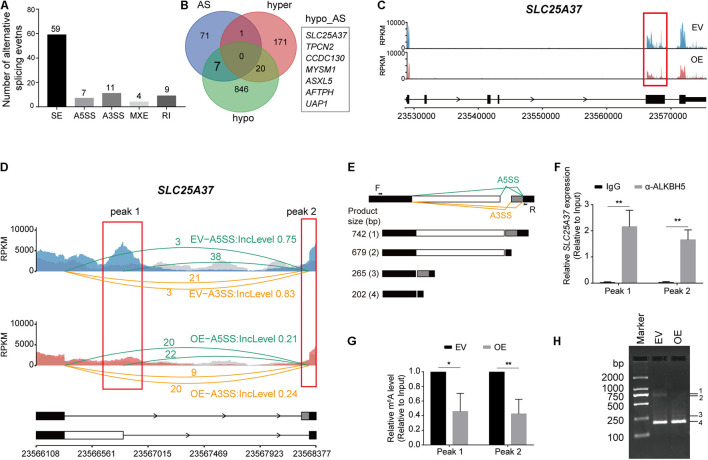
ALKBH5 regulates the alternative splicing events of *SLC25A37* RNA. **(A)** Statistics of the five types of alternative splicing events that are identified as significantly different in OE sample compared with EV sample. The differential alternative splicing events (FDR < 0.05) are identified by *rMATS*. **(B)** Venn plot exhibiting the intersects among the RNAs with hyper-, hypo-methylation peaks or differential alternative splicing events in OE sample. **(C)** Sequencing read density for input and m^6^A-IP samples along *SLC25A37* transcript. **(D)** Zoom-in of the hypo-methylated region on *SLC25A37* transcript. Sequencing read density of input and m^6^A-IP libraries along with exon junction read number were indicated. Inclusion levels of A3SS and A5SS events in both samples are recorded. Two concrete m^6^A peaks (Peak 1 and Peak 2) intersecting with alternative splicing sites are marked with red rectangles. **(E)** Diagram showing the alternative splicing formats of A5SS and A3SS on *SLC25A28* transcript. Black arrows denote the position of PCR primers. Below the figure exhibiting different splicing variants and their molecular weights of PCR products. **(F)** RIP-qPCR results showing the interaction between ALKBH5 protein and *SCL25A37* RNA at the two indicated regions in MIA PaCa-2 cells. **(G)** Results of m^6^A-RIP-qPCR showing the methylation changes at the two indicated regions between EV and OE samples. **(H)** PCR results showing the size and quantity of four types of transcripts in ALKBH5-overexpressing and control cells. *P < 0.05, **P < 0.01.

Besides RNA decay, RNA m^6^A methylation can also modulate AS of RNAs co-transcriptionally (Zhou et al., 2019). Thus, we utilized *rMATS* to detect the differential utilization of splicing sites for five types of AS events (A5SS, A3SS, MXE, RI, and SE) between the OE and EV samples. We detected varying numbers of AS events (Supplementary Table 8), which exhibited significant changes in the OE sample (Figure 5A). Considering that some of the differential AS events may be induced by aberrant methylation elicited by ALKBH5 overexpression, we analyzed the RNAs harboring both hypo-methylation and altered AS events after ALKBH5 overexpression (Figure 5B). Consequently, seven RNAs were filtered out. By combining with the total RNA-based m^6^A-seq data (Supplementary Tables 8, 9), we found that only *SLC25A37* exhibited significant changes in its methylation level and AS (Figure 5C and Supplementary Figure 5). We found that two types of AS (A5SS and A3SS) events occurred within the two hypo-methylated regions in *SLC25A37*, which generated four types of isoforms with different inclusion levels before and after ALKBH5 overexpression (Figures 5D,E). Isoform 4# corresponds to the canonical protein-coding transcript with normal length and function in mitochondrial iron delivery (Wang et al., 2011) (Supplementary Figure 6). In contrast, the splicing variants 2# and 3# were characterized by a retained intron, resulting from the A5SS and A3SS, respectively (Figure 5E). Notably, the sequence annotations in ENSEMBL (Howe et al., 2021) illuminated that the retained introns of isoforms 2# and 3# had a stop codon, which could generate a truncated mutant of 155 or 159 aa, respectively (Supplementary Figure 6). Moreover, an additional splicing variant 1# was also detected, which contained two pieces of retained introns and could have arisen from the concomitance of the A5SS and A3SS events. Similar to isoform 2#, it may be translated into a truncated protein mutant of 155 aa due to the presence of stop codon after the A5SS. We performed RIP-qPCR to detect the interaction between the ALKBH5 protein and *SLC25A37* RNA (Figure 5F), followed by m^6^A-IP-qPCR to confirm the effect of ALKBH5 on its m^6^A levels within two hypo-methylated regions (Figure 5G). Thereafter, we compared the RNA abundance of the four types of isoforms with PCR using a pair of primers capable of amplifying all the transcripts (Figure 5E). As shown in Figure 5H, we detected an increase in isoform 4#, corresponding to the decrease in isoform 1# and 2#, which was indicative of the presence of more functional transcripts of *SLC25A37* in the OE MIA PaCa-2 cells. Together, we show here that ALKBH5 regulates the RNA splicing of *SLC25A37* in m^6^A-dependent manner.

### ALKBH5 Modulates Iron Metabolism Regulators and Their Downstream Targets

**FIGURE 6:**
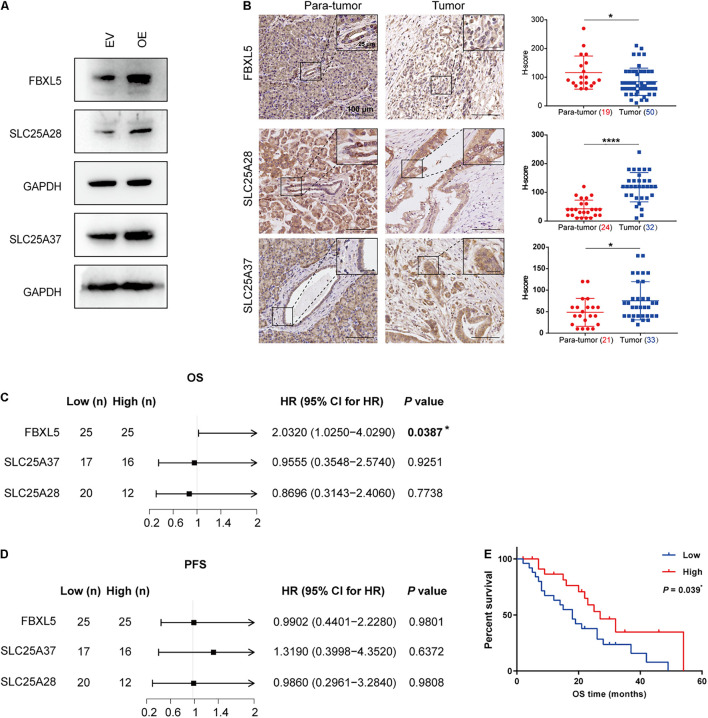
Downregulation of FBXL5 in PDAC predicts poor prognosis. **(A)** Western blot analysis showing the effect of ALKBH5 overexpression on FBXL5, SLC25A28, and SLC25A37. EV, empty vector; OE, ALKBH5-overexpressing cells. GAPDH is used as an internal control. **(B)** Representative images of immunohistochemical staining showing the expressions of FBXL5, SLC25A28, and SLC25A37 in para-tumor and tumor cells. Enlarged images in the box are shown in the upper right. Scatter plots showing the *H*-scores of each gene in para-tumor and tumor cells are shown in the right. Case numbers are shown in brackets. Scale bars, 100 or 25 μm. **(C,D)** Log-rank analysis of correlation between overall survival (OS) or progression-free survival (PFS) and the expression of FBXL5, SLC25A28 and SLC 25A37 in tumors. **(E)** Kaplan–Meier curves for OS of FBXL5 protein expression. *P < 0.05, ****P < 0.0001.

After identifying *FBXL5*, *SLC25A28*, and *SLC25A37* as substrate RNAs of ALKBH5, we further investigated the relevant mechanism associated with pancreatic cancer progression. In line with the effects of ALKBH5 on their RNA stabilities or AS patterns, we detected an elevation in the expressions of FBXL5 and SLC25A28 proteins in OE sample, as well as the functional protein form of SLC25A37 (Figure 6A). Furthermore, we examined their *in situ* protein expressions in tumor samples of PDAC patients. As expected, we observed significant decrease in the expression of FBXL5 protein (Figure 6B), which was in agreement with our results obtained *in vitro*. However, both SLC25A28 and SLC25A37 were upregulated in PDAC samples (Figure 6B). In addition, log-rank analysis showed that low expression of FBXL5 protein was associated with worse prognosis, while we failed to observe any correlation for SLC25A28 and SLC25A37 proteins (Figures 6C–E).

**FIGURE 7:**
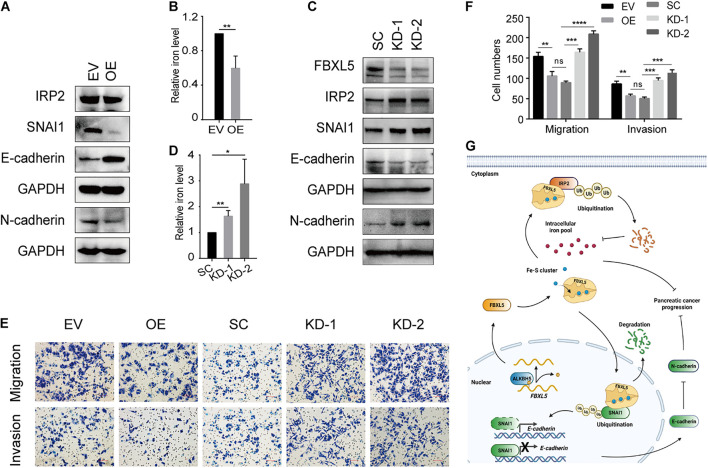
ALKBH5 protects against tumor progression in PDAC by targeting regulators of iron metabolism. **(A)** Western blot analysis showing the expressions of IRP2, SNAI1, E-cadherin, and N-cadherin in ALKBH5-overexpressing cells. EV, empty vector; OE, ALKBH5-overexpressing cells. GAPDH is used as an internal control. **(B)** Intracellular iron assay showing the effect of ALKBH5 overexpression on intracellular iron levels. **(C)** Western blot analysis showing the effect of *FBXL5* knockdown in rescuing the expression of IRP2, SNAI1, E-cadherin, and N-cadherin in ALKBH5-overexpressing cells. SC, scramble control; KD-1, *FBXL5* knockdown-1; KD-2, *FBXL5* knockdown-2. **(D)** Intracellular iron assay showing the effect of *FBXL5* knockdown in restoring intracellular iron levels in ALBKH5-overexpressing cells. **(E)** Cell migration and invasion assays of *FBXL5* knockdown in OE. **(F)** Statistical analysis for the result of transwell assays in **(E)**. Cell numbers per field were presented as the mean ± SD. *P < 0.05, **P < 0.01, ***P < 0.001, ****P < 0.0001. **(G)** Schematic diagram depicting the role of ALKBH5 in attenuating pancreatic tumorigenesis through targeting regulators of iron metabolism. (Created with BioRender.com).

FBXL5 protein plays a role in polyubiquitination and degradation of iron regulatory protein 2 (IRP2) and modulator of epithelial-mesenchymal transition (EMT) SNAI1 (Vinas-Castells et al., 2014; Wang H. et al., 2020). Accordingly, we found that ALKBH5 overexpression resulted in reduced expression of IRP2 protein (Figure 7A) and resultant reduction of intracellular iron levels (Figure 7B). ALKBH5-overexpression also led to downregulation of SNAI1, accompanied with upregulation of E-cadherin and downregulation of N-cadherin (Figure 7A). Intriguingly, we found that *FBXL5* knockdown rescued the expression of IRP2, SNAI1, and the two EMT markers (Figure 7C). Likewise, we observed a robust recovery of intracellular iron accumulation, as well as cell migratory and invasive abilities (Figures 7D–F). To validate the above findings, we examined the expression levels of IRP2 and SNAI1 proteins in PDAC samples. In line with downregulation of FBXL5 in tumor cells of PDAC, we found that both IRP2 and SNAI1 were upregulated (Supplementary Figure 7A). Notably, log-rank test showed that increased expression of IRP2 in tumor were associated with poor OS, while increased expression of SNAI1 was associated with poor PFS (Supplementary Figures 7B–E).

On the basis of the above identified functions of ALKBH5-FBXL5-IRP2/SNAI1 axis in pancreatic cancer cells, we proposed a working model for ALKBH5 in attenuating pancreatic tumorigenesis (Figure 7G). As an RNA m^6^A demethylase, ALKBH5 induces upregulation of FBXL5 via promoting its RNA stability. Subsequently, FBXL5 triggers the ubiquitination of IRP2 and SNAI1 proteins. IRP2 degradation would protect cells from intracellular iron overload, while downregulated SNAI1 suppresses the EMT process. Consequently, both of these actions contribute to impair tumor progression in ALKBH5-overexpressing cells. Taken together, our results reveal that ALKBH5 attenuates pancreatic cancer progression by targeting the regulators of iron metabolism.

## Discussion

The present study analyzed the clinical relevance of eight RNA m^6^A regulators based on their protein levels in the tumor and stromal cells in PDAC. We found that ALKBH5 was downregulated in the PDAC tumor cells and served as an independent, favorable prognostic marker. Mechanistically, we identified that ALKBH5 regulates the RNA stability of *FBXL5* and *SLC25A28*, as well as the AS of *SLC25A37*. Notably, we show here that upon ALKBH5 overexpression, the stabilized *FBXL5* further elicited the downregulation of the IRP2 and SNAI1 proteins, both of which are substrates ubiquitinated by FBXL5 and are crucial drivers of tumor progression.

The PDAC is a complex disease, by virtue of its heterogeneous cancer cell populations and extensive desmoplastic stroma (Neesse et al., 2019; Peng et al., 2019). The active crosstalk between the stromal and tumor cells is crucial in driving tumor progression (Hessmann et al., 2020). To date, the known functions of m^6^A regulators in PDAC tumorigenesis were limited to tumor cells only, which originate from normal pancreatic ductal epithelial cells and represent only a minority of the tissue mass in PDAC (Lee et al., 2019). Here we analyzed the expression levels of eight RNA m^6^A regulators in the tumor stroma cells to gain a better understanding of their distinctive characterization. Consequently, we observed a significant difference in the expressions of several RNA m^6^A regulators between the tumor cells and stromal cells. Particularly, the expression levels of the FTO and YTHDF1 proteins in the stroma were positively correlated with the OS or PFS of PDAC patients. These results suggest that m^6^A modification has an extensive influence on different components of PDAC tumor tissues, which warrants intensive investigation. It is noteworthy that although previous studies reported that the expressions of METTL3, METTL14, and WTAP exhibited negative correlation with the “patients’ OS” (Li et al., 2017; Xia et al., 2019; Wang M. et al., 2020), we did not obtain the same results in this study, probably owing to differences in the respective sizes of the study cohorts.

Intriguingly, out of the eight RNA m^6^A regulators analyzed in this study, only ALKBH5 served as an independent favorable prognostic factor, suggesting its massive impact in the progression of pancreatic cancer. Mounting evidences state that ALKBH5 plays versatile roles in various cancers. Concretely, ALKBH5 acts as an oncogene in glioblastoma (Zhang et al., 2017), acute myeloid leukemia (Shen et al., 2020), breast cancer (Zhang et al., 2016), and ovarian carcinoma (Jiang et al., 2020), while functioning as a tumor suppressor in lung cancer (Zhang et al., 2021), hepatocellular carcinoma (Chen et al., 2020), and osteosarcoma (Yuan et al., 2021). Moreover, it has also been reported that ALKBH5 exhibits tumor suppressive and chemo-sensitizing effects in pancreatic cancer cells (Guo et al., 2020; Tang et al., 2020). Nevertheless, different from our findings here, they identified *WIF-1* and *PER1* RNAs as key targets of ALKBH5, which further impacts the downstream WNT signaling and ATM-CHK2-P53/CC25C pathway, respectively (Guo et al., 2020; Tang et al., 2020). These studies imply that ALKBH5 disturbs myriad pathways to inhibit pancreatic cancer tumorigenesis.

In order to facilitate an in-depth scrutiny of the regulatory mechanism of ALKBH5 in PDAC, we exploited two types of m^6^A-seq analyses to identify its substrate RNAs, with respect to RNA stability or AS. We found that ALKBH5 regulated RNA decay of *FBXL5*, *SLC25A28*, and the AS of *SLC25A37*. Intriguingly, all of the three genes are involved in regulating iron metabolism. Iron is essential for diverse biological processes, while dysregulation of iron metabolism may lead to tumor progression and affects the response to therapy (Torti and Torti, 2020a). Although accumulating data implicate the association between m^6^A and tumor development, knowledge about the crosstalk between m^6^A and iron metabolism is extremely limited. FBXL5 is a member of the SCF ubiquitin ligase complex that specifically recognizes IRP2 (Wang H. et al., 2020), while FBXL5-IRP2 axis is integral to the control of iron metabolism (Moroishi et al., 2011). Accordingly, we found that in pancreatic cancer cells, ALKBH5-overexpression led to reduction of intracellular iron levels, and this could be restored via *FBXL5* knockdown. Thus, we deduce that the protective role of ALKBH5 in pancreatic cancer is closely related to FBXL5-mediated regulation of iron metabolism. Similarly, studies have reported that FBXL5 also plays a crucial role in tumor suppression in gastric (Wu et al., 2015) and liver cancers (Muto et al., 2019) via the maintenance of iron homeostasis. It has been reported that chronic exposure to excessive iron promotes EMT in pancreatic cancer and carcinogenesis (Bhutia et al., 2020). On the other hand, nuclear FBXL5 protein also functions as a ubiquitin ligase of SNAI1 (Vinas-Castells et al., 2014), which is a key modulator of the EMT and thus involved in multiple kinds of cancers (Moody et al., 2005; Carmichael et al., 2020; Chen R. et al., 2021). Therefore, FBXL5-induced degradation of SNAI1 protein and subsequent EMT changes also contributed to hinder tumor progression of PDAC. Combining the downregulation of FBXL5 in pancreatic cancer samples and its positive correlation with survival, our results indicate that FBXL5, as a downstream target of ALKBH5, plays a vital role in protecting against pancreatic cancer. Notably, significant downregulation of *Fbxl5* was also observed in the testis of *Alkbh5*-deficient mice, while the mechanism remains unknown (Zheng et al., 2013). It is worth to investigate whether ALKBH5-FBXL5 pathway is involved in multiple biological processes. Apart from our observation, a recent study found that YTHDF1 induced HPSCC tumorigenesis depended on iron metabolism (Ye et al., 2020). Given the significantly lower expression of YTHDF1 in the stromal cells and positive correlation with OS of patients with PDAC observed in this study, it will be interesting to determine whether stromal YTHDF1 also exerts an iron-metabolism dependent protective role in pancreatic cancer.

Previous studies reported that patients with erythropoietic protoporphyria (Wang et al., 2011) or myelodysplastic syndrome (Visconte et al., 2015) exhibited a significant decrease in the levels of the normal SLC25A37 isoform, accompanied with an increase in its abnormal isoform encoding a defective protein. Intriguingly, the same two types of isoforms were detected here in the pancreatic cell line. This may explain the reason for the elevated levels of normal SLC25A37 protein observed in the OE MIA PaCa-2 cells. Both SLC25A28 and SLC25A37 were essential for mitochondrial iron delivery and iron-sulfur (Fe-S) cluster synthesis (Kunji et al., 2020). Notably, FBXL5 protein harbors Fe-S clusters, which is indispensable for the recognition of IRP2 and promoting its degradation (Wang H. et al., 2020). Oppositely, defective Fe-S biogenesis caused ubiquitination and degradation of FBXL5, which in turn stabilized IRP2 (Rouault and Maio, 2020). Therefore, our results suggested that the ALKBH5-induced upregulation of SLC25A28 and SLC25A37 contributed to stabilization of FBXL5 protein. However, despite of positive regulation of ALKBH5 on SLC25A28 and SLC25A37 observed here, we found that these two proteins were overexpressed in the tumor cells of PDAC. We deduce that there may exist complicated mechanism in regulating their gene expressions and functions *in vivo*, which could not be fully characterized through cell line-based studies. For example, an *in vivo* studies by Li et al. (2018) reported that PINK1-PARK2 pathway mediated degradation of SLC25A37 and SLC25A28 proteins via autophagy-dependent pathway, thus preventing from mitochondria iron overload and tumorigenesis of PDAC. Similarly, the relative weaker correlation of RNA expression between *SLC25A28* and *ALKBH5* in tumor samples may also arise from other uncharacterized mechanisms in regulating SLC25A28. Therefore, the regulatory mechanism of SLC25A37 and SLC25A28, and their involvement in iron metabolism deserve intensive investigation.

## Conclusion

Our study reveals a previously uncharacterized mechanism of ALKBH5 in protecting against pancreatic cancer through modulating regulators of iron metabolism regulators and expanded our understanding of the association between m^6^A and iron homeostasis. Our results also underscored the multifaceted roles of m^6^A in pancreatic cancer, thus providing insight for the development of efficient therapeutic strategies for PDAC.

## Data Availability Statement

The raw m^6^A-seq data presented in this study have been deposited in the Genome Sequence Archive in BIG Data Center, Beijing Institute of Genomics (BIG), Chinese Academy of Sciences, under accession number HRA000878 that are accessible at https://bigd.big.ac.cn.

## Ethics Statement

The studies involving human participants were reviewed and approved by the Ethics Committee of the Peking Union Medical College Hospital (JS-1490). The patients/participants provided their written informed consent to participate in this study.

## Author Contributions

ZL and YN conceived this study and supervised the project. RH, ZZ, XL, and YF modified the methodology and performed the assays. LY analyzed the m^6^A-seq data. ZL and ZZ performed the clinical analysis. RH and LY wrote the manuscript. ZL, YN, and W-MT revised it. All authors read and approved the final manuscript.

## Conflict of Interest

The authors declare that the research was conducted in the absence of any commercial or financial relationships that could be construed as a potential conflict of interest.

## Publisher’s Note

All claims expressed in this article are solely those of the authors and do not necessarily represent those of their affiliated organizations, or those of the publisher, the editors and the reviewers. Any product that may be evaluated in this article, or claim that may be made by its manufacturer, is not guaranteed or endorsed by the publisher.

## Abbreviations

AML: acute myelogenous leukemia
AS: alternative splicing
A5SS: alternative 5 ′ splicing site
A3SS: alternative 3 ′ splicing site
BCA: bicinchoninic acid
DAB: diaminobenzidine
DMEM: Dulbecco’s modified Eagle’s medium
DMRs: differentially methylated regions
DTT: dithiothreitol
EDTA: ethylenediaminetetraacetic acid
EMT: epithelial-mesenchymal transition
EV: empty vector
FBS: fetal bovine serum
Fe-S cluster: iron-sulfur cluster
FDR: false discovery rate
Fe-S: iron-sulfur
FFPE, formalin-fixed: paraffin-embedded
GO: Gene Ontology
HPSCC: hypopharyngeal squamous cell carcinoma
HRP: horseradish peroxidase
H&E: hematoxylin and eosin
IHC: immunohistochemical
IJC: inclusion junction counts
IRP2: iron regulatory protein 2
MXE: mutually exclusive exons
m^6^A: *N*^6^-methyladenosine
m^6^A peaks: m^6^A-modified regions
m^6^A-seq: methylated RNA immunoprecipitation sequencing
OE: ALKBH5-overexpressing
OS: overall survival
PAAD: pancreatic adenocarcinoma
PCR: polymerase chain reaction
PDAC: pancreatic ductal adenocarcinoma
PFS: progression-free survival
PSI: percent spliced in
PVDF: (polyvinylidene fluoride)
qPCR: quantitative real-time polymerase chain reaction
RI: retained intron
RIP: RNA immunoprecipitation
RIPA: radioimmunoprecipitation assay buffer
rMATS: replicate multivariate analysis of transcript splicing
RNasin: RNase inhibitor
RPKM: reads per kilobase of transcript per million mapped reads
SD: standard deviation
SJC: skipping junction counts
SDS: sodium dodecyl sulfate
SE: skipped exon
TBS: Tris-buffered saline
TBST: Tris-buffered saline with Tween 20
TCGA: The Cancer Genome Atlas
TMA: tissue microarray.
