# Supplementary material for: RNA m6A Demethylase ALKBH5 Protects Against Pancreatic Ductal Adenocarcinoma via Targeting Regulators of Iron Metabolism
Source: Front Cell Dev Biol. 2021 Oct 18;9:724282. doi: 10.3389/fcell.2021.724282 (PMC8558440; doi:10.3389/fcell.2021.724282)
Supplement: Supplementary file 1 [file Data_Sheet_1.pdf]

Supplementary Materials

Supplementary Figures S1-S7 and figure legends.
Supplementary Table S1. List of antibodies used in this study.
Supplementary Table S2. List of primers used in this study.
Supplementary Tables S3-9 (see attached excel files).
Table S3. Summary of sequencing information, data processing, gene expression, and methylation analysis.
Table S4. Differential m^6A peaks between ALKBH5-overexpression (OE) and empty vector (EV) samples in poly(A) RNA-based m^6A-seq data and GO analysis of the protein-coding genes harboring differential methylation.
Table S5. Results of differential expression analysis in poly(A) RNA-based m^6A-seq data.
Table S6. Results of differential methylation analysis in total RNA-based m^6A-seq data.
Table S7. Results of differential expression analysis in total RNA-based m^6A-seq data.
Table S8. Results of differential alternative splicing analysis in poly(A) RNA-based m^6A-seq data.
Table S9. Results of differential alternative splicing analysis in total RNA-based m^6A-seq data.

Huang R. et al., Figure S1

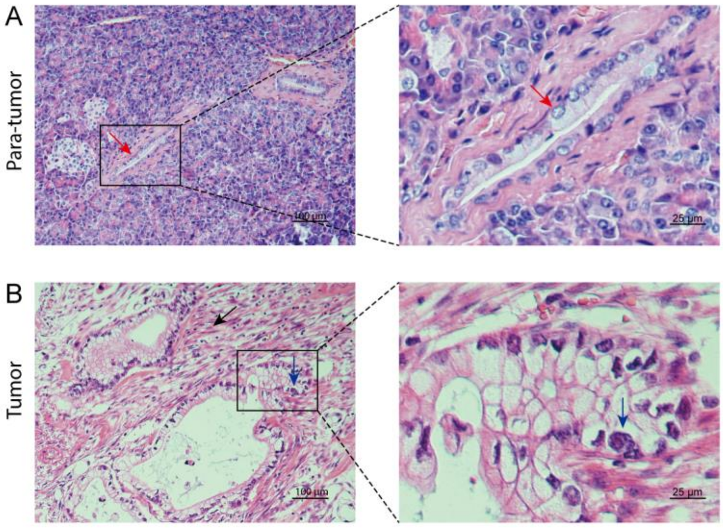

Supplementary Figure S1 Representative images of H&E analysis of para-tumor (A) and tumor (B) samples of PDAC patients. Red arrows indicate normal ductal epithelial cells, blue arrows indicate tumor cells, while black arrows indicate stromal cells. Enlarged images in the rectangle are shown in the right panel. Scale bars, 100 μm and 25 μm, respectively.

A

PFS-Tumor

	Low (n)	High (n)	HR (95% CI for HR)	*P*
METTL3	32	26	0.692 (0.320-1.500)	0.331
METTL14	32	25	2.164 (0.988-4.740)	0.330
WTAP	28	22	0.884 (0.374-2.089)	0.773
FTO	25	24	1.915 (0.824-4.455)	0.117
ALKBH5	29	27	1.607 (0.771-3.349)	0.178
YTHDF1	25	10	1.011 (0.327-3.125)	0.984
YTHDF2	30	26	1.542 (0.692-3.434)	0.275
YTHDF3	24	22	1.174 (0.509-2.708)	0.693

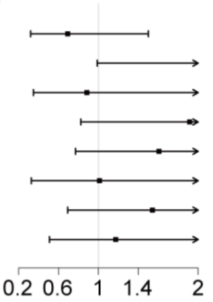

B

OS-Univariates

Characteristics	HR (95% CI for HR)	*P*
FTO Stroma (Low vs. High)	0.490 (0.245-0.981)	**0.044** *
YTHDF1 Stroma (Low vs. High)	0.432 (0.192-0.976)	**0.043** *

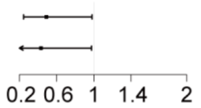

C

OS-Multivariates

Characteristics	HR (95% CI for HR)	*P*
Sex (Male vs. Female)	1.495 (0.615-3.637)	0.375
Position (Head vs. Body/Tail)	1.480 (0.501-4.375)	0.478
FTO Stroma (Low vs. High)	0.574 (0.207-1.590)	0.286
YTHDF1 Stroma (Low vs. High)	0.524 (0.207-1.326)	0.173

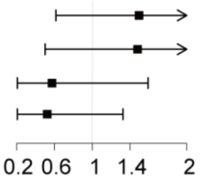

D

PFS-Univariates

Characteristics	HR (95% CI for HR)	*P*
Age (<65 vs. ≥65)	0.869 (0.423-1.786)	0.702
Sex (Male vs. Female)	1.120 (0.570-2.203)	0.742
Position (Head vs. Body/Tail)	1.586 (0.804-3.128)	0.183
LN Invasion (No vs. Yes)	2.849 (1.344-6.040)	**0.006** **
Neural Invasion (No vs. Yes)	1.066 (0.397-2.862)	0.899
Bile Invasion (No vs. Yes)	1.248 (0.572-2.723)	0.579
T Stage (T1/T2 vs. T3/T4)	0.816 (0.340-1.958)	0.648
N Stage (N0 vs. N1)	2.118 (1.040-4.314)	**0.039** *
M Stage (M0 vs. M1)	1.730 (0.654-4.577)	0.269
ALKBH5 Tumor (Low vs. High)	0.663 (0.317-1.387)	0.275
FTO Stroma (Low vs. High)	0.427 (0.188-0.971)	**0.042** *
YTHDF1 Stroma (Low vs. High)	0.405 (0.146-1.126)	0.083

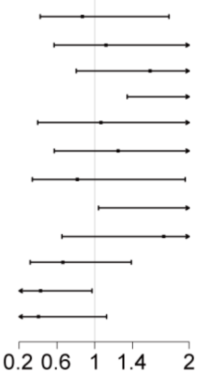

E

PFS-Multivariates

Characteristics	HR (95% CI for HR)	*P*
LN Invasion (No vs. Yes)	1.221 (0.257-5.797)	0.802
N Stage (N0 vs. N1)	2.043 (0.415-10.063)	0.380
FTO Stroma (Low vs. High)	0.411 (0.169-1.001)	0.050

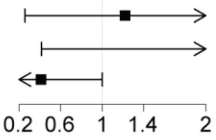

Supplementary Figure S2 Survival analysis for the expression levels of m^{6}A regulators and risk factors of PDAC. (A) Log-rank analysis showing the correlation between the expression of m^{6}A regulators in tumors and progression-free survival (PFS). (B, C) Cox regression analysis for overall survival (OS) in PDAC patients. (D, E) Cox regression analysis for PFS in PDAC patients. * $P < 0.05$, ** $P < 0.01$.

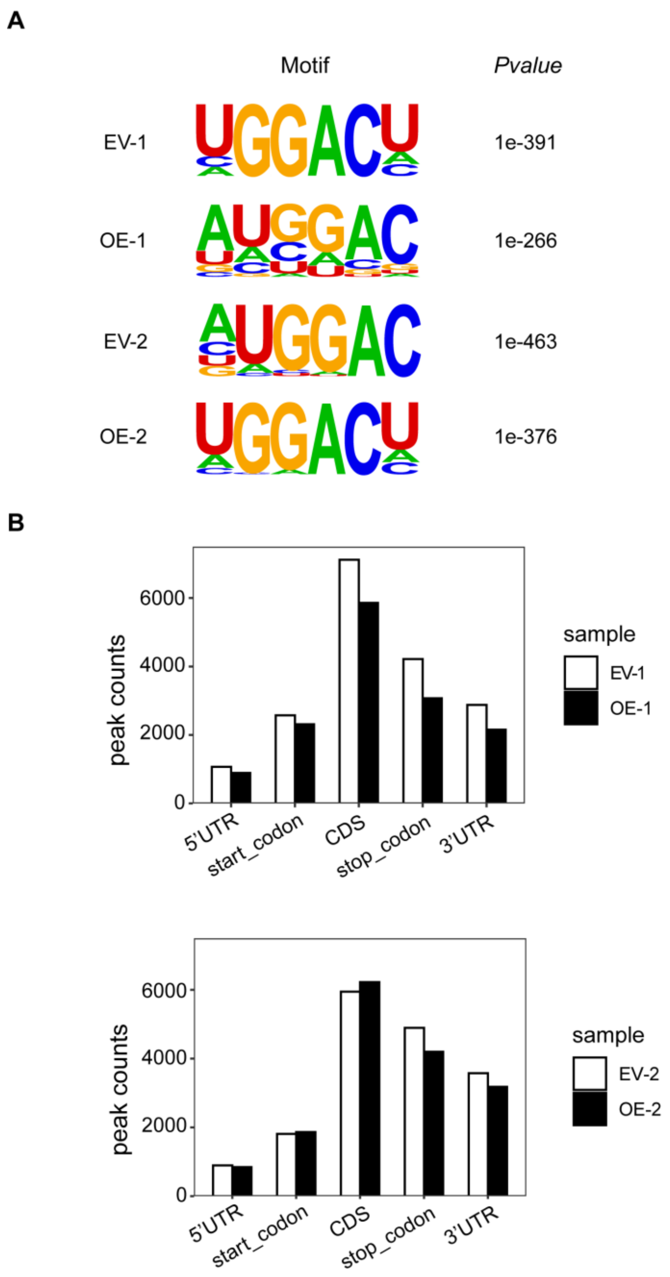

Supplementary Figure S3 Distribution of m^{6}A peaks in two sets of total RNA-based m^{6}A-seq in MIA PaCa-2 cells. (A) Top consensus sequences of all enriched motifs for m^{6}A peaks identified in empty control (EV) and ALBKH5 overexpression (OE) groups. (B) Bar graph showing the distribution of m^{6}A peak in different regions along the mRNA transcript.

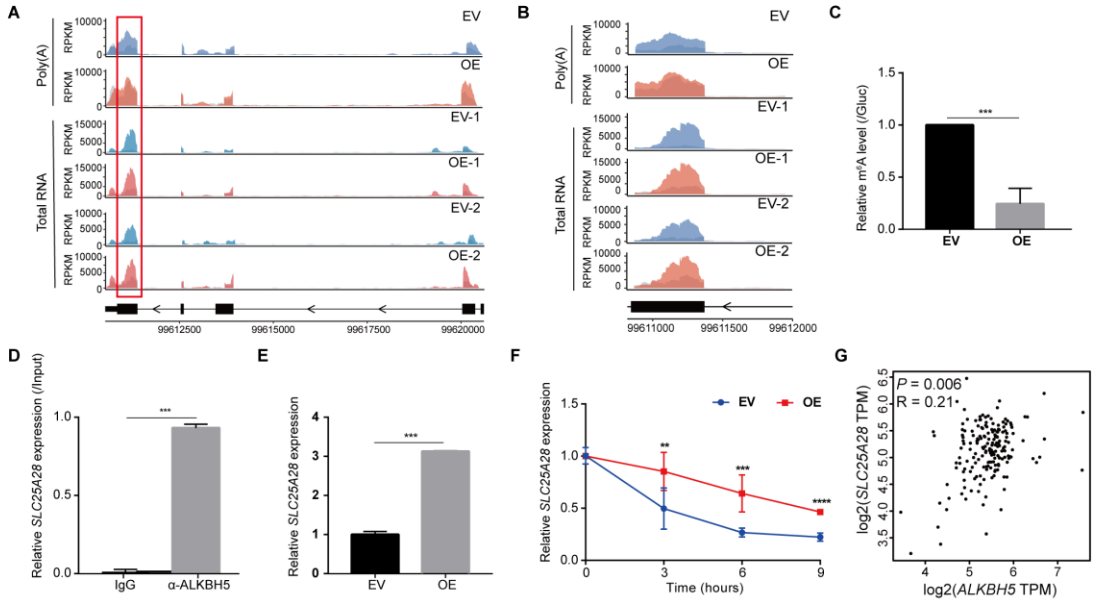

Supplementary Figure S4 ALKBH5 regulates the stability of *SLC25A28* RNA in MIA PaCa-2 cells. (A) Sequencing reads density for input and m^{6}A-IP samples along *SLC25A28* transcript in poly(A) RNA- and total RNA-based m^{6}A-seq data. Hypo-methylated peaks are indicated in red rectangles. Grey for input libraries, while blue (EV) or red (OE) for IP libraries. EV, empty vector; OE, ALKBH5 overexpression. X-axis, genomic coordinates; Y-axis, normalized number of reads. (B) Zoom-in of the hypomethylated region on *SLC25A28* transcript. (C) m^{6}A-IP-qPCR results showing the methylation changes of *SLC25A28* between EV and OE samples. (D) RIP-qPCR results showing the interaction between ALKBH5 protein and *SLC25A28* RNA. (E) RT-qPCR results showing the changes of *SLC25A28* expression between EV and OE samples. (F) Results of RNA stability assay showing the effect of ALKBH5 overexpression on the rate of *SLC25A28* RNA decay. (G) Correlation of RNA expression between *ALKBH5* and *SLC25A28* across 178 pancreatic adenocarcinoma (PAAD) samples in TCGA database. R, Pearson correlation coefficient. ** $P < 0.01$, *** $P < 0.001$, **** $P < 0.0001$.

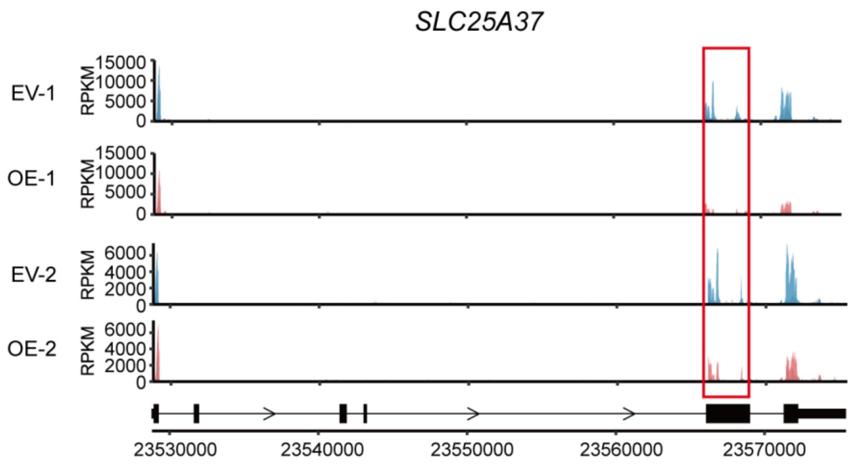

Supplementary Figure S5 Reads density plots showing m^{6}A methylation level of *SLC25A37* identified in two sets of total RNA-based m^{6}A-seq data. Sequencing reads density for input and m^{6}A-IP samples along SLC25A28 transcript in poly(A) RNA- and total RNA-based m^{6}A-seq data. Hypo-methylated peaks are indicated in red rectangles. Grey for input libraries, while blue (EV) or red (OE) for IP libraries. EV, empty vector; OE, ALKBH5 overexpression. X-axis, genomic coordinates; Y-axis, normalized number of reads.

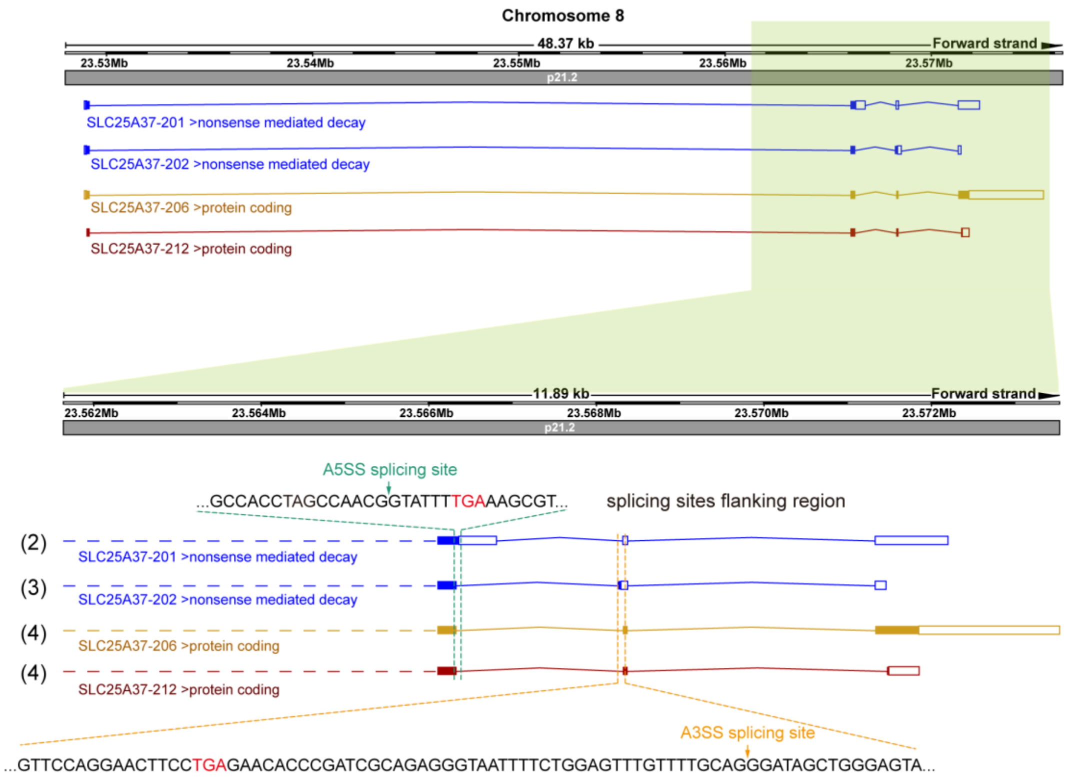

Supplementary Figure S6 Diagram showing *SLC25A37* isoforms resulting from alternative splicing. Overall display (upper panel) and zoom-in (lower panel) of the alternatively spliced regions on *SLC25A37* transcript were shown. (2), (3), and (4) correspond to the PCR products detected in Figure 5E and 5H. Part of cDNA sequences of the splicing variants (2) and (3) are shown to highlight the positions of splicing sites and the stop codon TGA present in the retained introns.

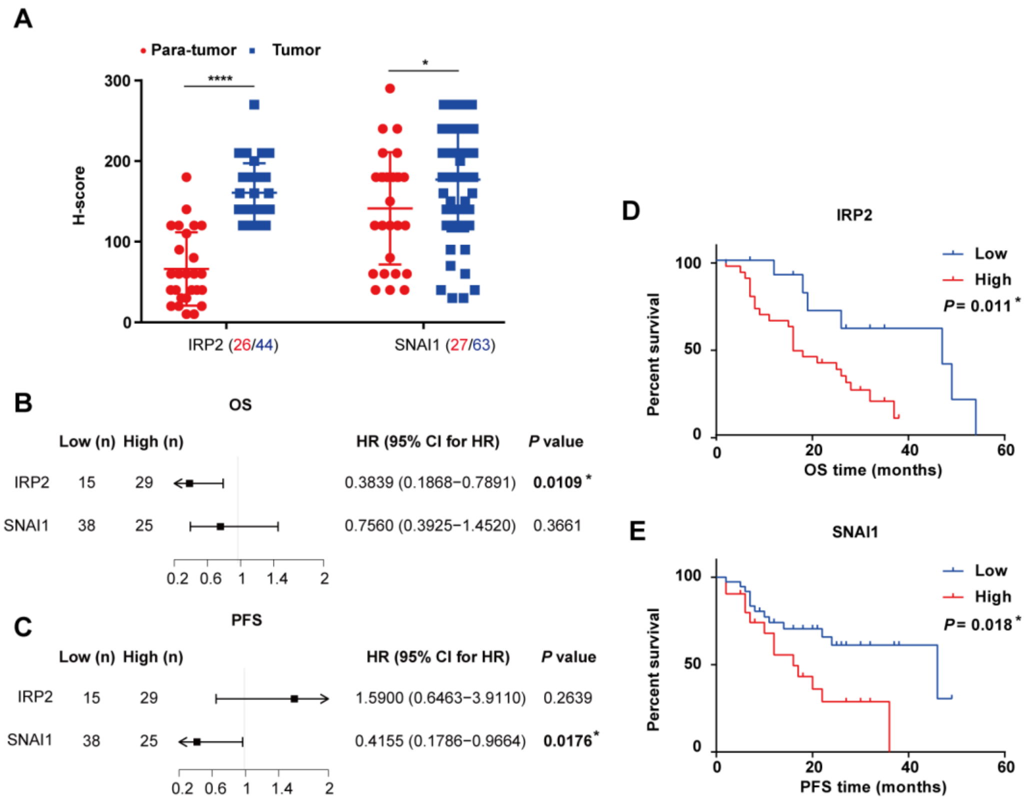

Supplementary Figure S7 *In situ* protein expression and survival analysis of IRP2 and SNAI1 in tumor samples of PDAC. (A) Scatter plots showing the H-scores of IRP2 and SNAI1 in para-tumor and tumor cells. Case numbers are shown in brackets, red for para-tumor and blue for tumor. (B, C) Log-rank analysis showing the correlation between overall survival (OS) or progression-free survival (PFS) and the expression of IRP2 and SNAI1 in tumor cells. (D, E) Kaplan-Meier survival curves for OS or PFS of IRP2 and SNAI1 protein expression. * $P < 0.05$, **** $P < 0.0001$.

Table S1 List of antibodies used in this study.

Antibody Name	Source	Catalog No.	Application	Dilution ratio
anti-GAPDH	ZSGB-BIO	TA-08	WB	1: 1000
Anti-β-Actin	Santa cruz	Sc-47778	WB	1: 1000
anti-ALKBH5	Proteintech	16837-1-AP	WB, RIP, IHC	WB: 1:1000, RIP: variable, IHC: 1:500
anti-FTO	Proteintech	27226-1-AP	IHC	IHC: 1:500
anti-METTL3	Proteintech	15073-1-AP	IHC	IHC: 1:500
anti-METTL14	Abcam	ab220030	IHC	IHC: 1:500
anti-WTAP	Proteintech	60188-1-lg	IHC	IHC: 1:500
anti-YTHDF1	Proteintech	17479-1-AP	IHC	IHC: 1:500
anti-YTHDF2	MBL	RN123PW	IHC	IHC: 1:100
anti-YTHDF3	Abcam	ab103328	IHC	IHC: 1:500
Rabbit polyclonal to m^6A	Abcam	ab151230	m^6A-IP	Variable
Rabbit Flag mAb (D6W5B)	CST	14793S	m^6A-IP	Variable
Rabbit (DA1E) mAb IgG	CST	3900	m^6A-IP	Variable
Mouse Anti-rabbit IgG	CST	5127	RIP	Variable
Anti-SLC25A37	Proteintech	26469-1-AP	WB	1:500
Anti-SLC25A28	Bioss	Bs-7157R	WB	1:500
Anti-FBXL5	Solarbio	K107646P	WB	1:5000
Anti-IRP2	Proteintech	23829-1-AP	WB	1:400
Anti-SNAI1	CST	3879	WB	1:1000
Anti-SNAI1	Solarbio	K003876P	IHC	1:100
Anti-E-cadherin	CST	3195	WB	1:1000
Anti-N-cadherin	CST	13116	WB	1:1000
Peroxidase-Conjugated Goat anti-Rabbit IgG (H+L)	ZSGB-BIO	ZB-2301	WB	1:1000
Peroxidase-Conjugated Goat anti-Mouse IgG (H+L)	ZSGB-BIO	ZB-2305	WB	1:1000

Table S2 List of primers used in this study.

Primers	Sequence (5′→3′)
Gluc F	CGACATTCCTGAGATTCCTGG
Gluc R	TTGAGCAGGTCAGAACACTG
Cluc F	GCTTCAACATCACCGTCATTG
Cluc R	CACAGAGGCCAGAGATCATTC
FBXL5 F	ACCACAATACGCAAAGGCTG
FBXL5 R	GTTGTTCCACCTCTGGTTGT
SLC25A28 F	CAAACGTCCAGTGGGGTTGT
SLC25A28 R	TAACCCCCAGAGACGGTACA
18S F	CATTCGAACGTCTGCCCTAT
18S R	GTTTCTCAGGCTCCCTCTCC
SLC25A37 Peak 1 F	GAGTCCAGATCCCAAAGCCC
SLC25A37 Peak 1 R	TGATCATGACGTTGACGCCT
SLC25A37 Peak 2 F	GAGAACACCCGATCGCAGAG
SLC25A37 Peak 2 R	TCATTACCGCATCGTGGAGC
SLC25A37 AS F	TCTACGGAGCCCTCAAGAAA
SLC25A37 AS R	TGGCCATACTCCCAGCTATC

F: forward primer
R: reverse primer
